# Impact of optical coherence tomography scan direction on the reliability of peripapillary retinal nerve fiber layer measurements

**DOI:** 10.1371/journal.pone.0247670

**Published:** 2021-02-22

**Authors:** Abinaya Priya Venkataraman, Josefine Andersson, Lina Fivelsdal, Maria Nilsson, Alberto Domínguez-Vicent

**Affiliations:** Division of Eye and Vision, Department of Clinical Neuroscience, Karolinska Institute, Stockholm, Sweden; University of Melbourne, AUSTRALIA

## Abstract

**Purpose:**

To evaluate the intradevice repeatability and agreement for peripapillary retinal nerve fiber layer (pRNFL) measurements in healthy eyes with two different scan directions and two different number of B scans.

**Methods:**

pRNFL was measured with a spectral domain optical coherence tomography on 54 healthy participants. Three-dimensional optic disc scans (6 mm x 6 mm) were performed on the right eye of the participants. Two repeated scans were performed in four different settings: H1: Horizontal scan with 512 A-scans x 96 B-scans; H2: Horizontal scan with 512 A-scans x 128 B-scans; V1: Vertical scan with 512 A-scans x 96 B-scans; V2: Vertical scan with 512 A-scans x 128 B-scans. The pRNFL thickness was evaluated in twelve clock-hour sector in a circle of 3.45 mm diameter centred at the optic disc. Repeatability and agreement were assessed with within subject standard deviation (Sw) and Bland-Altman test respectively.

**Results:**

The repeatability of pRNFL measurements varied depending on the scan direction and sectors. The repeatability for the horizontal sectors were better with H1 and H2, with sector 9 having the best Sw (< 3 μm). The repeatability for the vertical sectors were better with V1 and V2 with sector 5 and 9 having the best Sw (< 4 μm). The repeatability with vertical scan was more symmetric among the sectors than with horizontal scans. The repeatability metrics of the sectors did not vary much between H1 and H2 (difference < 2 μm) and between V1 and V2 (difference < 3.2 μm). Comparing horizontal and vertical scans, the vertical sectors had larger limits of agreement of about 45 μm.

**Conclusion:**

The reliability of the pRNFL thickness measurements is dependent on the direction of the scan and independent on the numbers of B-scans. Vertical scans for pRNFL gives more homogeneous repeatability across the different sectors.

## Introduction

Clinical and research practice have been revolutionized after the introduction of Optical Coherence Tomography (OCT) [1], since it allowed the acquisition of in-vivo cross-sectional images of the retina and choroid. This technology allows the objective measurements of the retinal layers thicknesses, and it is used for diagnosing and monitoring retinal pathologies [2, 3], and glaucoma [4–6]. Since the retina is part of the central nervous system, OCT has been also used in neurological studies involving multiple sclerosis [7, 8], Parkinson [9] or Alzheimer [10] among others.

Previous studies have shown that abnormal changes in the retinal layers, such as retinal nerve fiber layer (RNFL) or ganglion cell layer (GCL), precede to visual function defects [11]. For example, glaucoma is known to cause a reduction in the GCL and RNFL thickness, and previous studies have proven that GCL and RNFL loss precedes visual field defects [12, 13]. The ability of OCT in detecting damage to the RNFL, macular GCL, and optic nerve head in both pre-perimetric and perimetric glaucoma is well documented in the literature [14]. The RNFL thickness reductions in the inferior and superior regions around the optic nerve are used as a clinical biomarker in glaucoma diagnosis [15].

Nowadays, there are several OCT instruments available for clinical use, each includes its own segmentation algorithm software to delineate the retinal layers. Precision studies are needed to know how consistent the measurements of the retinal layers are, since the precision depends on the segmentation algorithm, scan resolution, scan direction, and acquisition time [16–19]. Modern OCT devices allow the clinician to customize the scan settings, such as the number of A- and B-scans, scan length, or scan direction. Previous studies that assessed the intra-device repeatability of OCT devices to measure the peripapillary RNFL (pRNFL) thickness have shown that the repeatability varies between the vertical and horizontal sectors, and the vertical sector measurements have lower repeatability [19–21]. This is detrimental for the usefulness of OCT in glaucoma diagnosis as vertical sector damage is shown to be an important clinical marker [15].

In a previous study [19] from our group, we have reported that the repeatability for macular thickness measurements in different sectors is dependent on the scan direction. The vertical sectors showed better repeatability with vertical scans and the horizontal sectors showed better repeatability with horizontal scans. In the present study, we wanted to evaluate if the same pattern can be observed even for the pRNFL measurements. We evaluated the intradevice repeatability for pRNFL measurements in healthy eyes with two different scan directions and two different number of B scans. The results from this study could help to define measurement protocols that can provide more reliable pRNFL measurements.

## Material and methods

### Subjects

The study protocol adhered to the tenets of the Declaration of Helsinki and was approved by the Regional Ethical Committee (Regionala etikpröningsnämden, Stockholm 2011/874-31/2). A total of 54 healthy subjects aged between 18 and 30 years participated in this study. Written informed consent was obtained after explaining about the purpose, nature, and the possible consequences of the study. The inclusion criteria for participation were best corrected visual acuity (BCVA) 0.0 logMAR, refractive error ranging between ±5 D in sphere and smaller than 3D in cylinder, intraocular pressure below 21 mmHg, no history of ocular diseases or surgery. The initial screening measurements included complete ocular and medical history, BCVA, refraction, intraocular pressure with non-contact tonometry, slit lamp biomicroscopy, and undilated fundus photography.

### Measurements

All participants underwent OCT imaging with the HOCT-1F (Huvitz, South Korea), which is a spectral domain OCT with an axial resolution around 6 to 7 μm, transverse resolution of 20 μm, and acquisition rate of 68,000 A-scans per second. Three-dimensional optic disc scans (6 mm x 6 mm) were performed on the right eye of the participants. Two repeated scans were performed in four different settings: H1: Horizontal scan with 512 A-scans x 96 B-scans; H2: Horizontal scan with 512 A-scans x 128 B-scans; V1: Vertical scan with 512 A-scans x 96 B-scans; V2: Vertical scan with 512 A-scans x 128 B-scans. In case of poor fixation, subject blink or signal strength less than 6 (out of 10), the scans were repeated. The measurements were performed with sufficient breaks in between. All OCT measurements were performed by two experienced examiners.

**Fig 1:**
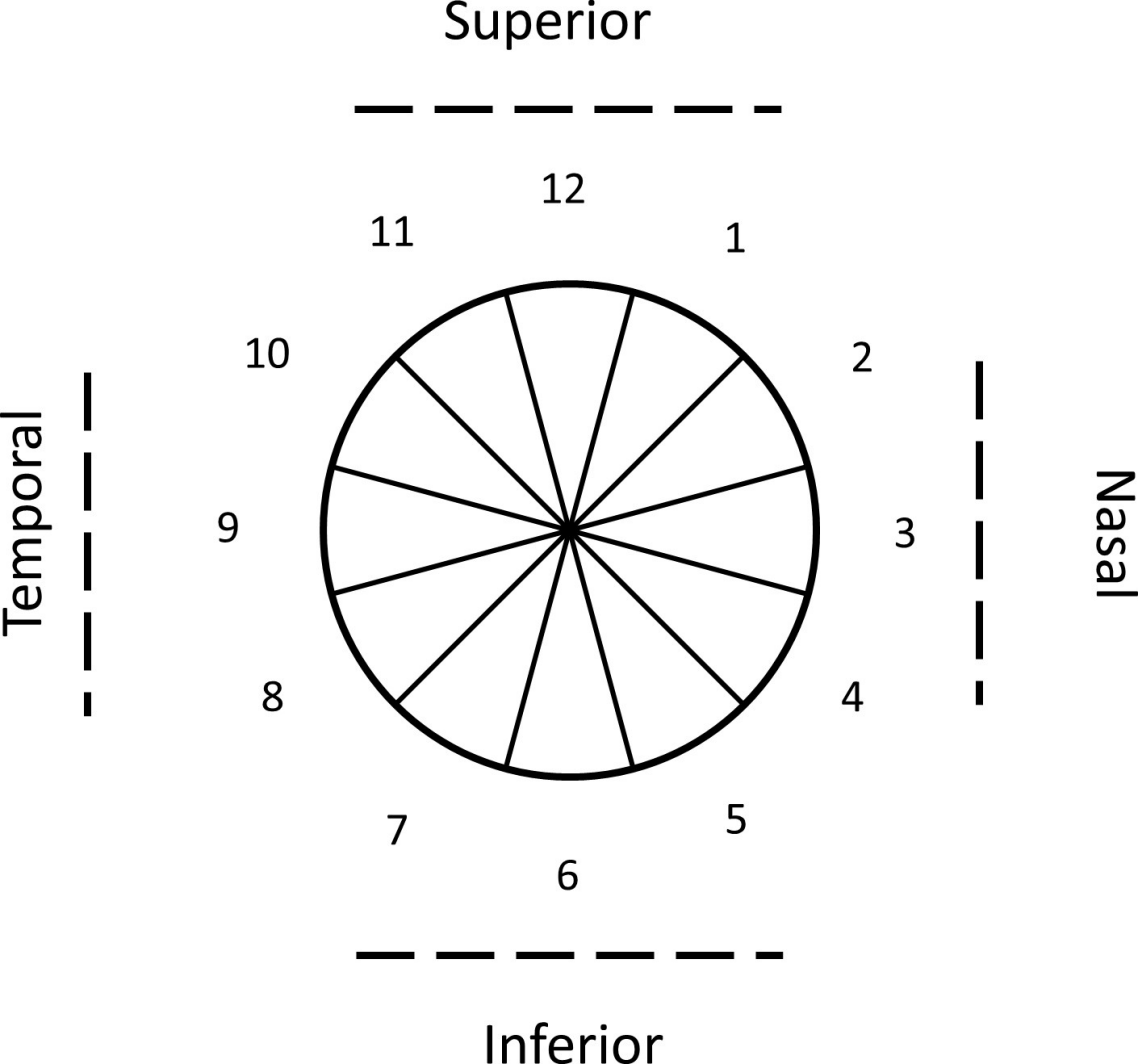
Schematic representation of the peripapillary retinal nerve fiber layer thickness measurement sectors. The numbers represent the clock hour positions.

The pRNFL thickness (from inner limiting membrane to retinal nerve fiber layer, ILM-RNFL) around the optic nerve head was obtained using the automated segmentation algorithm from the OCT instrument. No manual adjustments of the segmentation were performed. The pRNFL was then evaluated in twelve clock-hour sectors in a circle of 3.45 mm diameter centred at the optic disc. Fig 1 shows the schematic representation of the clock positions and the corresponding sectors.

### Statistical analysis

The baseline demographics of the observations and participants are summarized with descriptive statistics. The repeatability metrics for the two repeated measurements were the within subject standard deviation (Sw) and repeatability limits. The Sw, which represents the repeatability of the measurements, was calculated with a one-way analysis of variance with the subject as a factor [22]. The repeatability limit was calculated as $1.96∙\sqrt{2}∙S_{w}$, and it represents the expected limits that 95% of the measurements should be within. Sw and repeatability limits were calculated for each of the four scan settings. The Bland-Altman test [23] for repeated measurements was used to analyze the agreement between horizontal and vertical scans.

## Results

**Fig 2:**
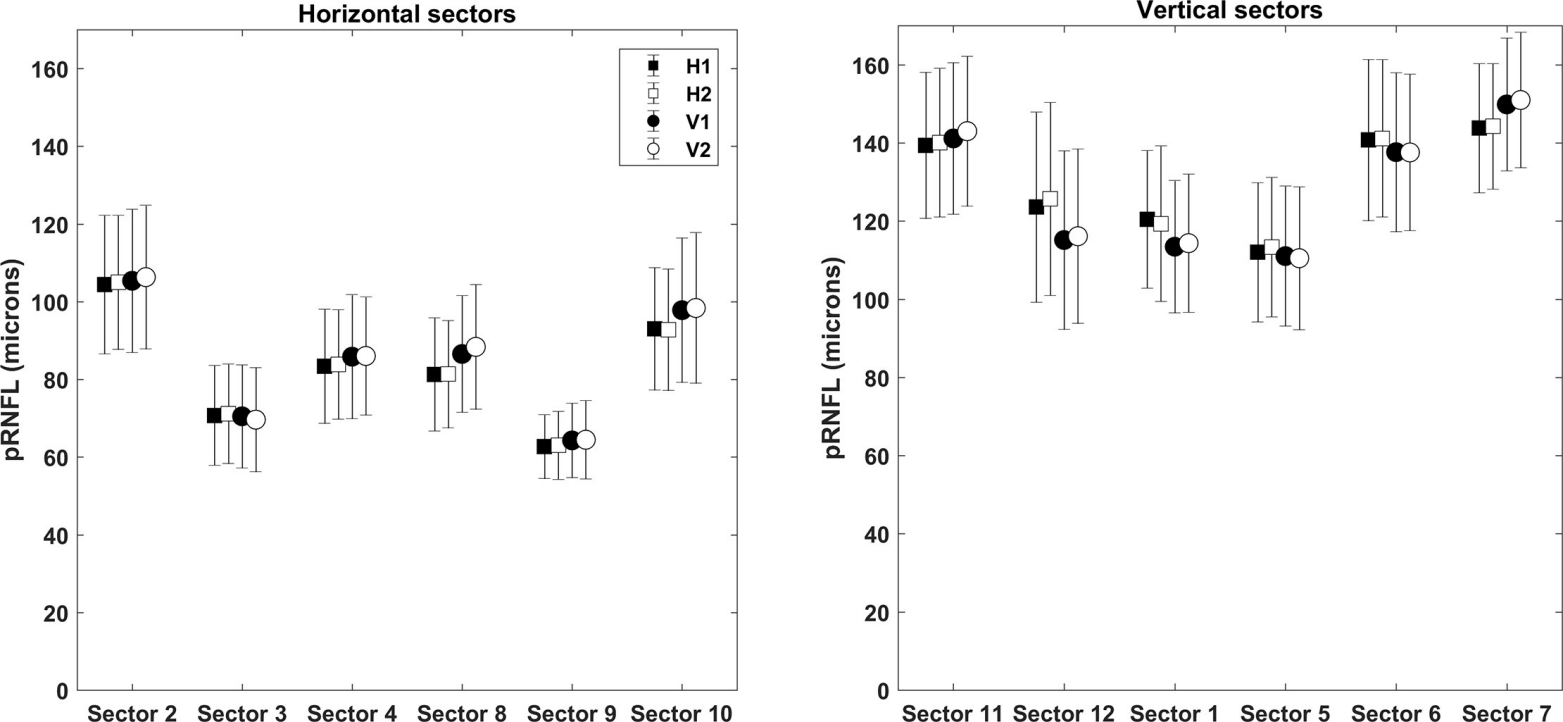
The average peripapillary retinal nerve fiber layer thicknesses in the six horizontal sectors (clock sectors 8–10 and 2–4) and six vertical sectors (clock sectors 11–1 and 5–7) from four different scan settings (H1, H2, V1 and V2). H1 and V1 represents scan setting with 512 A-scans x 96 B-scans in horizontal and vertical scanning respectively. H2 and V2 represents scan setting with 512 A-scans x 128 B-scans in horizontal and vertical scanning respectively.

The mean age of the participants was 24.6 ± 2.8 years. The average pRNFL thicknesses in the six horizontal sectors (clock sectors 8–10 and 2–4) and six vertical sectors (clock sectors 11–1 and 5–7) from four different scan settings are shown in Fig 2. In all scan settings, the pRNFL was thicker in vertical sectors compared to horizontal sectors as expected.

**Fig 3:**
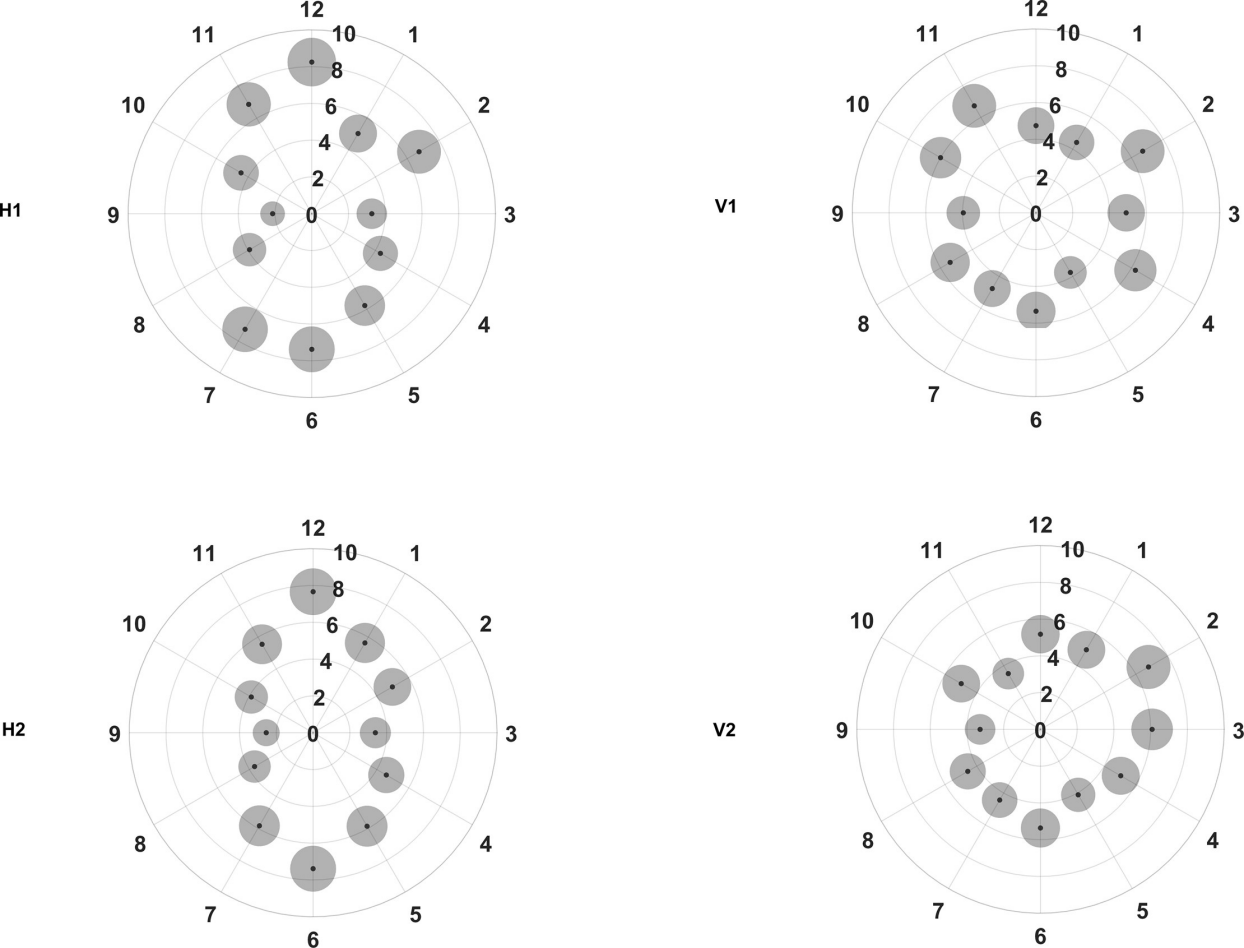
Repeatability for the peripapillary retinal nerve fiber layer thickness in different clock hour sectors for the four different scan settings (H1, H2, V1 and V2, refer to Fig 2 legends for specifications). The small black dots represent the within subject standard deviation and the grey circles surrounding each black point represent the repeatability limits, where the areas are scaled by a factor of 30 points for visualization purposes.

Fig 3 shows the repeatability of the two consecutive measurements of pRNFL in each scan setting. Overall, the repeatability metrics were good with Sw values not exceeding 8.5 μm in any of the sectors. However, the repeatability values in different sectors varied depending on the scan direction. The Sw values were distributed asymmetrically with horizontal scan direction, whereas the distribution was more symmetric with vertical scan direction. The Sw for the vertical sectors was larger with horizontal scan direction (H1 and H2) than with vertical scan direction (V1 and V2). However, the opposite tendency can be noted for the horizontal sectors.

It can be seen from Fig 3 that the Sw values in H1 and H2 are distributed like a vertically elongated ellipse. The best repeatability was seen in sector 9 with both H1 and H2, with Sw value of 2.1 and 2.6 μm respectively. Both H1 and H2 showed the worst repeatability in sector 12, with Sw value of 8.2 and 7.7 μm respectively. The repeatability metrics for the same sectors did not vary much between H1 and H2 with the maximum difference being less than 2 μm.

Even with the vertical scan settings (V1 and V2); the repeatability metrics varied among the different sectors. However, the variations in the repeatability metrics were not as large as that of horizontal scan settings as shown in Fig 3. The best repeatability was seen in sector 5 with V1 and in sector 9 with V2, with Sw values of 3.7 and 3.3 μm respectively. The worst repeatability was seen for sectors 2 and 11 with V1 (Sw of 6.7 μm) and in sector 2 with V2 (Sw of 6.8 μm). Except in sector 11, V1 and V2 showed similar repeatability values. Comparing V1 and V2, the repeatability metrics for the same sector were similar except for sector 11, where the Sw with V2 was 3.2 μm less than with V1.

The mean difference and limits of agreement between the horizontal and vertical scan settings are shown in Table 1. The limits of agreement interval for H1 and V1 were similar to that of H2 and V2 in all the sectors. The best agreement was seen for sector 9, where the limits of agreement were 23 and 29 μm for scan settings 1 and 2 respectively. The widest agreement limits were seen for sectors 6, 11 and 12, where the limits were more than 45 μm. On average, the horizontal sectors had 10 μm shorter intervals than vertical sectors.

**Table 1:** Comparison of peripapillary retinal nerve fiber layer thickness between horizontal and vertical scan settings in different sectors.

	Quadrants	Clock sectors	Mean difference (in microns)		Average limits of agreement interval	
			(Limits of agreement)			
			H1 and V1	H2 and V2	H1 and V1	H2 and V2
**Horizontal Sectors**	Nasal	Sector 2	-0.98	-1.35	34.87	34.96
			(-23.13 to 21.18)	(-19.4 to 16.7)		
		Sector 3	0.24	1.51		
			(-13.73 to 14.21)	(-17.11 to 20.14)		
		Sector 4	-2.46	-2.19		
			(-18.64 to 13.72)	(-17.95 to 13.58)		
	Temporal	Sector 8	-5.25	-7.02	29.27	31.01
			(-20.25 to 9.76)	(-22.97 to 8.92)		
		Sector 9	-1.62	-1.43		
			(-13.18 to 9.93)	(-14.48 to 11.63)		
		Sector 10	-4.79	-5.62		
			(-22.14 to 12.56)	(-23.12 to 11.89)		
**Vertical Sectors**	Superior	Sector 11	-1.78	-2.92	42.66	41.94
			(-25.19 to 21.64)	(-23.95 to 18.11)		
		Sector 12	8.4	9.56		
			(-15.08 to 31.88)	(-14.19 to 33.31)		
		Sector 1	7.01	4.98		
			(-10.09 to 24.1)	(-13.15 to 23.11)		
	Inferior	Sector 5	0.96	2.83	42.04	39.01
			(-16.21 to 18.14)	(-13.39 to 19.06)		
		Sector 6	3.09	3.62		
			(-22.9 to 29.07)	(-21.03 to 28.27)		
		Sector 7	-6.07	-6.73		
			(-25.98 to 13.83)	(-24.38 to 10.92)		

## Discussion

In the present study, we evaluated the differences in repeatability metrics for pRNFL measurements with horizontal and vertical scans. We also evaluated if the repeatability is dependent on the number of B scans in both scan directions. The comparisons between the thickness measurements under different settings were also assessed. There are three main findings from this study. First, the repeatability of pRNFL measurements in different sectors is dependent on the scan direction. Second, the agreement between horizontal and vertical scans also varies depending on the sector measured. Finally, both the repeatability and agreement did not vary much with the number of B-scans.

### Repeatability with different scan settings

The repeatability metrics were heterogeneous among the sectors with both horizontal and vertical scans. With the horizontal scans, the heterogeneity was more evident as the vertical sectors had larger repeatability limits compared to the horizontal sectors. Simply put, pRNFL measurements in horizontal sectors are more repeatable than vertical sectors with horizontal scans. This tendency is seen irrespective of the number of B scans (H1 and H2). Previous studies have also shown that the repeatability varies among the sectors [19–21]. Depending on the metric used to specify repeatability, the interpretation varies even within the same study [21, 24]. In these studies, the repeatability is reported in terms of both intraclass correlation coefficient (ICC) and Sw. Though Sw of vertical and horizontal sectors were not similar, the ICC was similar. Based on the Sw values, the horizontal sectors have better repeatability [14, 20, 24] compared to the vertical sectors and the opposite can be seen for the coefficient of variation. The coefficient of variation is directly dependent on the Sw value and inversely dependent on the actual thickness value. Sectors with thin pRNFL will have a larger coefficient of variation and this could explain the difference in repeatability metrics and how it is interpreted. The horizontal sectors have thinner pRNFL, so even when the Sw is smaller, the coefficient of variation could be larger, and the opposite applies for vertical sectors.

With vertical scans, we observed that the repeatability improved on the vertical sectors improved but worsened on the horizontal sectors. This reduces the heterogeneity of the repeatability among the different sectors in both V1 and V2. The maximum different in the Sw among the different sectors with horizontal scans was twice that of the vertical scans. Fig 3 shows that the repeatability is more homogenous with vertical scans than the horizontal scans. In the diagnosis and follow up of glaucoma patients, OCT plays a major role. In particular, pRNFL measurements in the vertical sectors are shown to have a better diagnostic capability in differentiating glaucoma eyes [25, 26]. It is important to have a good and homogenous repeatability among all the sectors. Though the scan resolution and segmentation algorithms are different in different OCT instruments, the tendency found in the present study could be seen in other OCT instruments as well. The scan protocol varies between instruments, both in terms of number of scans and scan direction (vertical, horizontal, circular and radial). Based on the current results, performing a vertical scan on the optic nerve head would be more appropriate given that the Sw is not varying much among the sectors. Alternatively, we can use radial scans or combine horizontal and vertical scans to get the best possible precision in every sector.

### Comparison of horizontal and vertical scans

Comparing the pRNFL thickness measured with horizontal and vertical scans, the horizontal sectors show better agreement compared to the vertical sectors in both scan setting (1 and 2). Comparison of macular thickness measurements with horizontal and vertical scan directions has been reported previously [19, 27, 28]. To our knowledge, this is the first study that compared the influence of different scan directions on the pRNFL measurements.

It has been shown that the blood vessels are the major reason for inconsistencies in the segmentation algorithm and affect pRNFL measurements [29, 30]. This can have more impact in the vertical sectors, where major blood vessels are present. It is also reported that blood vessels can contribute to the intersubject variability in the pRNFL profile measurements. This variability is shown to be larger in glaucoma eyes compared to healthy eyes [29]. Our findings suggest that the effect of blood vessels on the segmentation algorithm can be minimized with vertical scan direction than with horizontal scan direction.

Based on the repeatability metrics, we can estimate the minimum number of scans needed to ensure a specific measurement tolerance, MT (*MT* = (1.96∙*Sw*)/√*N*) for N number of measurements according to the ISO standards [31, 32]. In order to have MT as small as the axial resolution of the instrument (7 μm), 5 repeated measurements will be needed for horizontal scans whereas 4 repeated measurements will be needed for vertical scans, independently of the number of B scans. These estimates are based on the sector that had the worst Sw. From clinical perspective, it is not always possible to perform repeated measurements. A better alternative would be to combine horizontal and vertical scans. Another option would be to perform radial and/or circular scans, however it has been reported that line scans provide smaller bias and imprecision compared to radial and circular scans [33]. The findings and suggestions from the present study are based on young healthy eyes. It is reported previously that age is not a factor that influences the presence of artifacts in OCT imaging [34]. Several previous studies have reported that the intersession variability of the pRNFL measurements is more in glaucoma eyes than in healthy eyes [35–37]. Hence, we can expect that the findings from the present study can also be applicable for older participants and for eyes with glaucoma.

In conclusion, the reliability of the pRNFL thickness measurements is dependent on the direction of the scan and independent on the numbers of B-scans examined in this study. Vertical scans for pRNFL gives more homogeneous repeatability across the different sectors.

## Supporting information

S1 Data (XLSX)
